# Oral Neuroma in a Dog: A Case Report

**DOI:** 10.1177/08987564261428801

**Published:** 2026-03-06

**Authors:** Lisete Vieira, Fernanda Seixas, João Machado, Maria Frada, Carlos Viegas, Maria dos Anjos Pires, João Filipe Requicha

**Affiliations:** 1 Department of Veterinary Sciences, 56066 University of Trás-os-Montes e Alto Douro (UTAD), Vila Real, Portugal; 2 Veterinary Teaching Hospital, UTAD, Vila Real, Portugal; 3 Animal and Veterinary Research Center (CECAV) - Associate Laboratory for Animal and Veterinary Sciences (AL4AnimalS), UTAD, Vila Real, Portugal; 4 Vasco da Gama Research Centre, University School Vasco da Gama, Coimbra, Portugal

**Keywords:** *Canis familiaris*, incidental finding, lip, neuroma, oral cavity

## Abstract

A neuroma is a tangled, circumscribed, non-neoplastic mass of neuronal tissue formed after axonal injury and consists of a mixture of fibroblasts, Schwann cells and axons. Traumatic neuroma is the most commonly recognized neuroma lesion in humans and can occur at any site in the body where a nerve is traumatized. A 15-year-old female Miniature Pinscher was presented with periodontal disease. A lump on the right side of the face was found, corresponding to a solitary cylindrical mass on the mucosal surface of the lower lip. Dental radiographs showed no lysis of the mandibular bone. After an inconclusive cytological examination, the mass was surgically removed, fixed in 10% formalin, embedded in paraffin, and routinely stained for light microscopy. Macroscopically, a whitish, firm, filiform mass measuring 2.5 × 0.5 × 0.3 cm was found. Microscopically, the mass was poorly defined and consisted of spindle-shaped cells forming bands, bundles, and sometimes coiled structures, resembling nerves, surrounded by fibrosis. The cells were monomorphic, but an area of increased cellularity and nuclear atypia was observed. Mitotic figures, Verocay bodies, or Antoni A and B patterns were not found. The mass was surrounded by oral epithelium, mucinous glands, and skeletal muscle. There is much confusion in veterinary medicine regarding the nomenclature of peripheral nerve sheath tumors, that is, schwannomas, neurofibromas, perineuromas, traumatic neuroma, and malignant tumors of the peripheral nerve sheath. This lesion had histological features consistent with a neuroma, an unusual entity in the canine oral cavity.

## Introduction

A neuroma is a tangled, circumscribed proliferation of a peripheral nerve, usually reparative/reactive rather than a true neoplasm.^1–7^ It is a rare pathological condition of the peripheral nervous system consisting of a localized but disorganized proliferation of injured nerve elements.^8–10^ It appears as a palpable nodular mass^8,11^ at the proximal end of an injured peripheral nerve,^9,12^ more often in small nerves than in large nerves.^4^ Rarely, it can occur in the central nervous system, with reports of it being in the spinal cord^7,9^ and brainstem.^7^

It represents an exaggerated reactive hyperplasia that is an attempt to repair the nerve,^2,8,9,12^ and is composed of an admixture of fibroblasts, Schwann cells and axons.^2,3,5,6,9,12^ Traumatic neuroma is the most frequently recognized neuroma lesion in humans and is well-known to occur in the extremities, in other parts of the body where a peripheral nerve is traumatized, as well as in the oral cavity.^3,9,13^ In the oral cavity, the most commonly affected sites include the tongue, the lip,^1,13–15^ mainly the lower lip,^15^ and the mental nerve,^1,3,13–15^ although intraosseous lesions have also been reported.^13,15^ Tooth extraction,^3,15,16^ removal of the dental pulp tissue and incision and drainage of a dental abscess^2,3^ have been associated with trigeminal traumatic neuroma.^2^ Injury can also be iatrogenic due to nerve injury caused by the use of scalpels, chisels or drills^2^ or from a local anesthetic injection, as well as from an accident^13^ or a burn.^2^

In veterinary medicine, this abnormal regenerative tumor-like lesion is more common at tail dock sites,^5,17^ mainly in piglets, but also in dogs^12^ and lambs.^12,17^ It can also develop at other amputation sites, as well as neurectomy and fracture sites.^5,12^ Neuroma has been documented after posterior digital neurectomy in horses and after beak amputation in chickens.^12^ In dogs, there are published cases of neuroma in the tail,^12^ in the eye,^6^ and in the spinal cord.^9^

## Case Description

**Figure 1.**
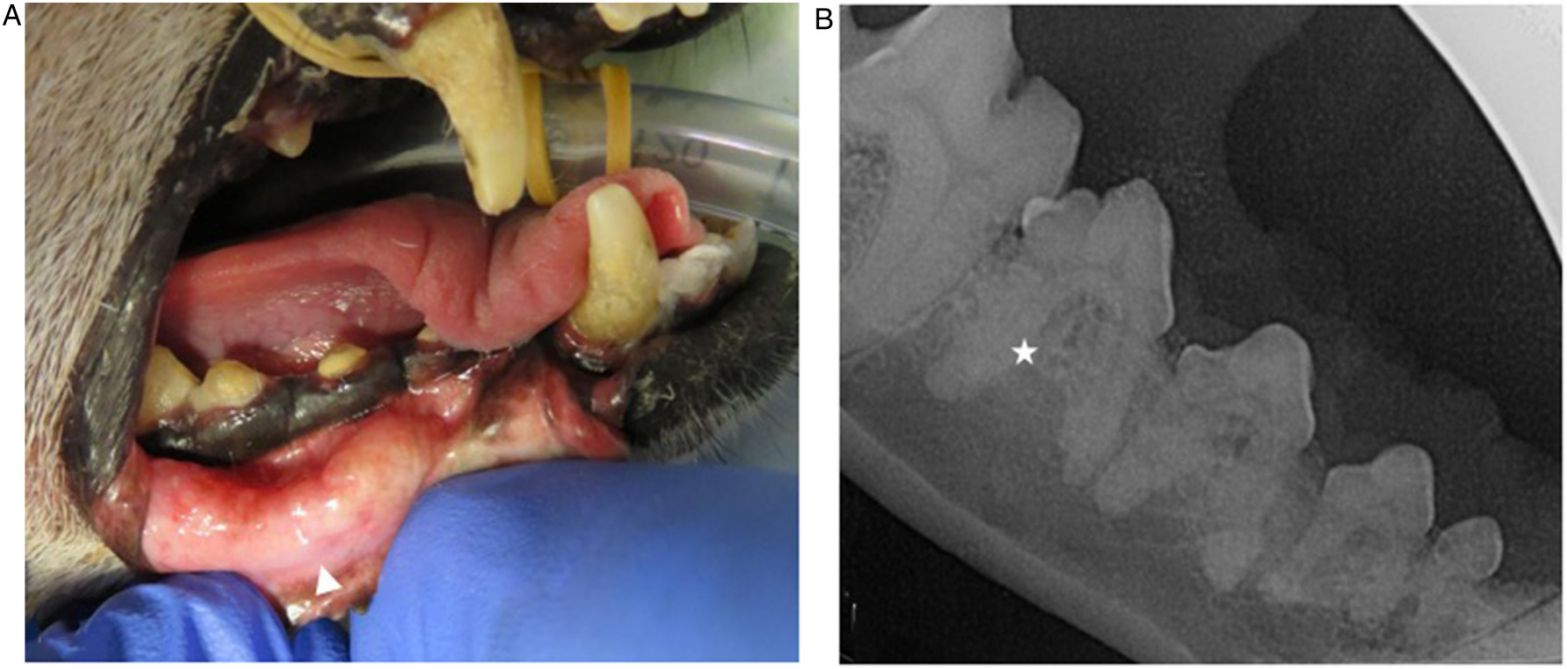
(A) Clinical Presentation of the Right Labial Swelling (Arrowhead) and (B) Dental Radiograph of the Adjacent Right Mandibular Fourth Premolar Tooth (Star).

A 15-year-old female Miniature Pinscher dog was presented at the Veterinary Teaching Hospital of the University of Trás-os-Montes e Alto Douro, Vila Real, Portugal, with periodontal disease. On clinical examination, a lump was noted on the right aspect of the oral cavity, consisting of a solitary, cylindrical mass on the mucosal surface of the lower lip (Figure 1A). No specific history of known maxillofacial or lip trauma was documented in this animal.

The animal was anesthetized with a premedication of fentanyl^a^ (1 µg/kg) and midazolam^b^ (0.05 mg/kg), both administered intravenously (IV). Anesthesia was induced with propofol IV, titrated to effect, to facilitate endotracheal intubation, and maintained with volatile isoflurane. During the procedure, methadone^c^ (0.1 mg/kg) intramuscularly and ampicillin^d^ (20 mg/kg) IV were administered. Fluid therapy with Ringer's lactate solution was given at a rate of 5 mL/kg/h, and the animal was continuously monitored.

**Figure 2.**
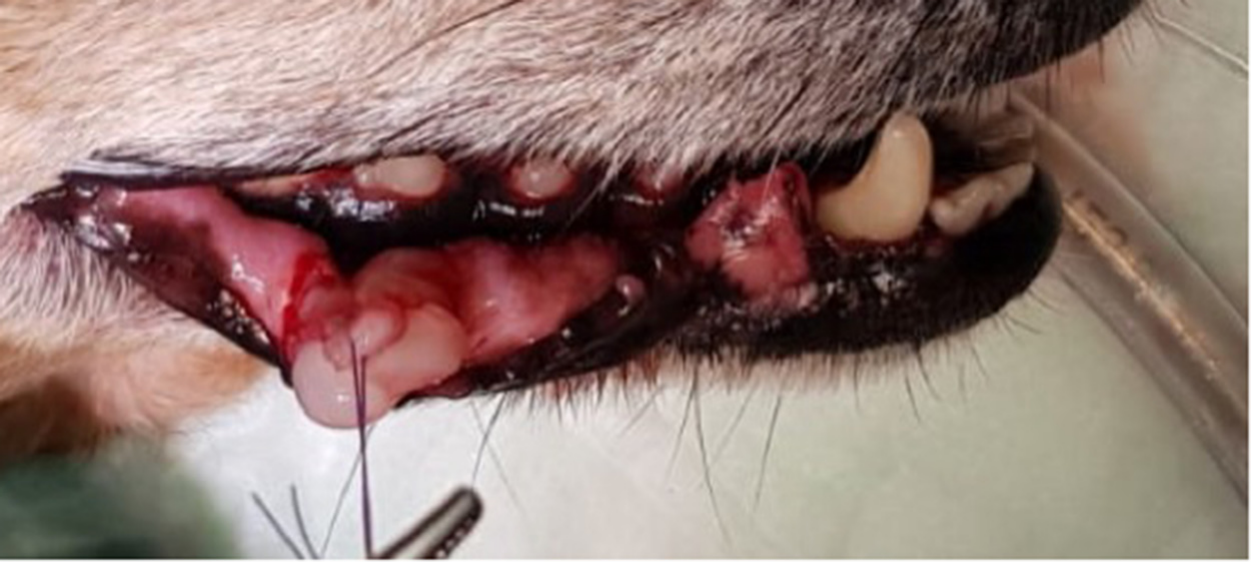
Site of Excisional Biopsy of the Labial Mass. Note Stay Suture in Place.

A comprehensive oral health assessment and treatment (COHAT) was performed, revealing mild periodontal disease and radiographic evidence of radicular ankylosis (Figure 1B). No preoperative evaluation, including advanced imaging, was performed to predetermine the surgical margins. Macroscopically, the lesion appeared as a well-defined, whitish, firm, filiform mass measuring 2.5 × 0.5 × 0.3 cm. An excisional biopsy was performed, following its clinically visible borders without additional safety margins (Figure 2). Post-procedure, meloxicam^e^ (0.1 mg/kg) per os (PO) once a day for 5 days and tramadol^f^ (3 mg/kg) PO twice a day for 3 days were prescribed. Additionally, the dog was advised to be fed small meals of soft food. One week later, wound healing was progressing well, with no signs of oral disease, and the dog was eating dry food without difficulty.

The excised sample was fixed in 10% neutral-buffered formalin^g^, embedded in paraffin, and processed for light microscopy using routine techniques. Sections were stained with hematoxylin and eosin (H&E)^h^, van Gieson, and reticulin stains.

**Figure 3.**
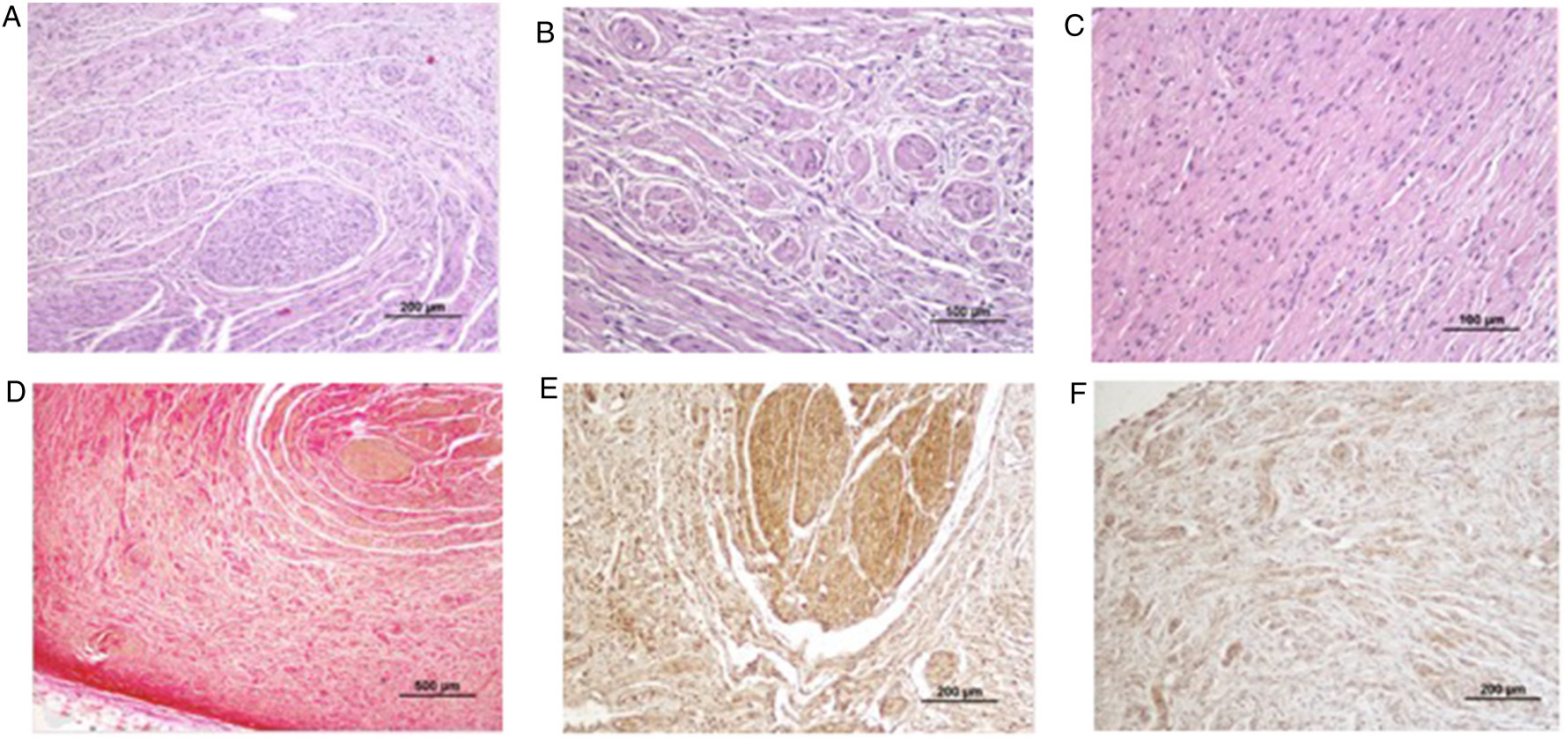
Histomicrographs Showing Spindle Cells Forming Bands and Bundles, Mimicking Nerves, Surrounded by Fibrosis (A) 200 µm (Hematoxylin and Eosin Stain - H&E); (B) 500 µm (H&E); (C) 100 µm (H&E). (D) Perineural Fibrosis (500µm) (Van Gieson’s Stain) Showing Strong Cytoplasmic Immunopositivity of Nervous Structures to Immunohistochemical (IHC) Marker/Stains; (E) Neuron-Specific Enolase (NSE) (200 µm); and (F) Vimentin (VIM) (200 µm).

Microscopically, the mass was poorly defined and consisted of spindle-shaped cells forming bands, bundles, and sometimes coiled structures, mimicking irregularly arranged small nerves, surrounded by dense fibrous tissue (Figure 3A to C). Cells were monomorphic, but an area of increased cellularity and nuclear atypia was observed. Neither mitotic figures nor Verocay bodies nor cells arranged in a cellular palisaded pattern (Antoni type A) nor a loose paucicellular pattern (Antoni type B) were found. The mass was occasionally surrounded by oral epithelium, mucinous glands, and skeletal muscle, but the lesion was not fully present in the surgical margin. Van Gieson staining confirmed the presence of abundant and dense stromal collagen fibers in the masses surrounding nerve structures, and reticulin staining revealed a sparse and delicate network of stromal reticulin fibers surrounding individual fusiform cells (Figure 3D).

Immunohistochemistry was performed using a polymeric labelling kit^i^ according to the manufacturer's instructions. Sections were incubated with vimentin^j^ (1:50) and neuron-specific enolase^k^ (NSE, 1:200), and immunoexpression was visualized using 3,3′-diaminobenzidine solution. The cells were positive for NSE (Figure 3E) and vimentin (Figure 3F). These findings led to the diagnosis of a neuroma, an unusual condition found in the oral cavity of dogs.

At a follow-up examination one year after the diagnosis, the dog was in good condition, alert, with no clinical signs, and there was no recurrence of the oral mass.

## Discussion

Traumatic neuromas are essentially failed attempts at regeneration in which the distal stump of a partially or completely transected or otherwise disrupted nerve is too separated to establish continuity^9–12^ or is blocked by tissue destruction and inflammatory response,^10,15,18^ resulting in an abnormal mass of disorganized proliferating axons in a background of Schwann cells, perineural cells, and epineurial, perineurial and endoneurial connective tissue.^6,9,17^

During regeneration, the endings of afferent sensory fibers become trapped within scar tissue and generate spontaneous or abnormal neuronal firing.^17^ It has long been proposed that a combination of nerve repair, wound contraction, and scar formation (which are a part of the wound healing process), and the associated neuronal proliferation contribute to the formation of traumatic neuroma.^2,19^

Traumatic neuromas are slow-growing lesions,^7^ usually < 5 cm,^10,11,18^ characterized by the presence of pain, burning, or paresthesia.^2,3,11,13,17^ Palpable swelling and pain on palpation under pressure are signs of traumatic neuroma.^3^ Although pain has traditionally been considered a feature of this lesion, some studies suggest that only some traumatic neuromas are painful^3,13,16,17^ and this pain can be either intermittent or constant.^13^ In the present clinical case, the animal did not show any clinical signs of pain or discomfort when grasping and chewing food or when the oral cavity was manipulated, indicating that the injury was an incidental finding during the performance of the comprehensive oral health assessment and treatment.

Macroscopically, the neuroma usually presents as a slow-growing, white, oval, and firm mass.^11,18^ Microscopically, it is a non-encapsulated lesion, containing a large number of randomly arranged nerve fascicles, within a densely collagenous and fibroblastic stroma^1^ with low blood vessel density.^18^

Traumatic neuromas are histologically somewhat similar in appearance to schwannomas but are more highly differentiated.^6^ Myelination of these axons is generally sparse compared with that of an intact nerve.^9,10^ A chronic inflammatory cell infiltrate may be seen in a minority of cases, particularly in symptomatic cases.^16,17^

A traumatic neuroma of the oral mucosa presents as a smooth-surfaced nodular mass. It is associated with altered nerve sensation or pain, which may be spontaneous or provoked by manipulation of the lesion.^15^

Several studies have demonstrated the role of ultrasound as an ancillary diagnostic tool in the field of traumatic peripheral nerve injury, namely by confirming and/or excluding the presence of suspected injuries.^8^

The treatment of choice is surgical excision,^1,10–13,15^ with minimal manipulation and destruction of nerve fibers.^13^ Most such lesions do not recur, although some may recur or be associated with persistent pain at the site.^15^ Once they occur, the optimal treatment is controversial. Any attempt at surgical repair or reconstruction will, by definition, result in a new neuroma, while symptom reduction is the goal of treatment.^10^

In veterinary medicine, neuroma has only recently been explicitly included in the classification of pseudotumoral lesions of the nerve sheath, despite occasional published case reports in different species.^5^ The lesion's location on the mucosal surface of the lower lip, in close contact with the dental arcade, supports the plausibility of chronic microtrauma as a triggering factor for this suspected oral traumatic neuroma. The diagnosis was based on the lesion's characteristic morphology, immunohistochemical profile and absence of neoplastic features, consistent with a reactive proliferative response of peripheral nerve elements.

## Conclusion

The histological and immunohistochemical features of this case were consistent with a neuroma, an unusual and poorly understood entity in the canine oral cavity. Chronic trauma between teeth and adjacent soft tissues could have been the triggering factor for the development of this unusual lesion. To the authors’ knowledge, this is the first report of an oral traumatic neuroma in a dog.

## Materials

- Fentadon 50 µg/mL, Dechra, Barcelona, Spain.
- Midazolam 15 mg/3 mL, Hikma, Terrugum, Portugal.
- Insistor 10 mg/mL, Vetviva Richter GmbH, Wels, Austria.
- Ampicillin 500 mg, Labesfal, Santiago de Besteiros, Portugal.
- Meloxidyl, Ceva Santé Animal, Libourne, France.
- Tramadol Retard, Grünenthal, Algés, Portugal.
- Panreac, Barcelona, Spain.
- QPath, Fontenay-sous-Bois, France.
- NovoLink Polymer Detection System, Leica Biosystems, Milton Keynes, UK.
- Agilent Technologies, Cheshire, UK.
- Zymed Thermo Fisher Scientific, Porto Salvo, Portugal.
